# Inadvertent insertion of a nasogastric tube into both main bronchi of an awake patient: a case report

**DOI:** 10.1186/1757-1626-2-6914

**Published:** 2009-05-27

**Authors:** Yohanna M Takwoingi

**Affiliations:** Department of Otolaryngology - Head and Neck Surgery, City HospitalDudley Road, Birmingham B18 7QH, UK

## Abstract

The use of nasogastric tube is desirable for the short-term administration of calories when oral feeding is not possible. Although the insertion of nasogastric tubes has been described as being easy this is not without risks. An unusual case of malpositioning of a fine bore nasogastric tube into both main bronchi in a patient that was awake is reported. Respiratory complications of misplaced nasogastric tubes and the importance of a check chest x-ray following tube placement are discussed.

## Introduction

Enteral feeding has gained acceptance in the nutritional support of various groups of patients. The use of enteral route has been facilitated by the development of highly flexible nasogastric tubes. The insertion of nasogastric tubes has been described as being easy, requiring little training and usually uneventful [1]. Wire stylets are commonly used to stiffen these soft, flexible tubes for blind insertion and also to aid in identifying the position of the tube on x-ray examination following insertion. However, the stylets are also sometimes responsible for guiding the tubes into the tracheobronchial tree [2]. A case of malpositioning of a fine bore nasogastric tube into both main bronchi detected on chest x-ray in an otherwise asymptomatic patient is reported.

## Case presentation

**Figure 1.**
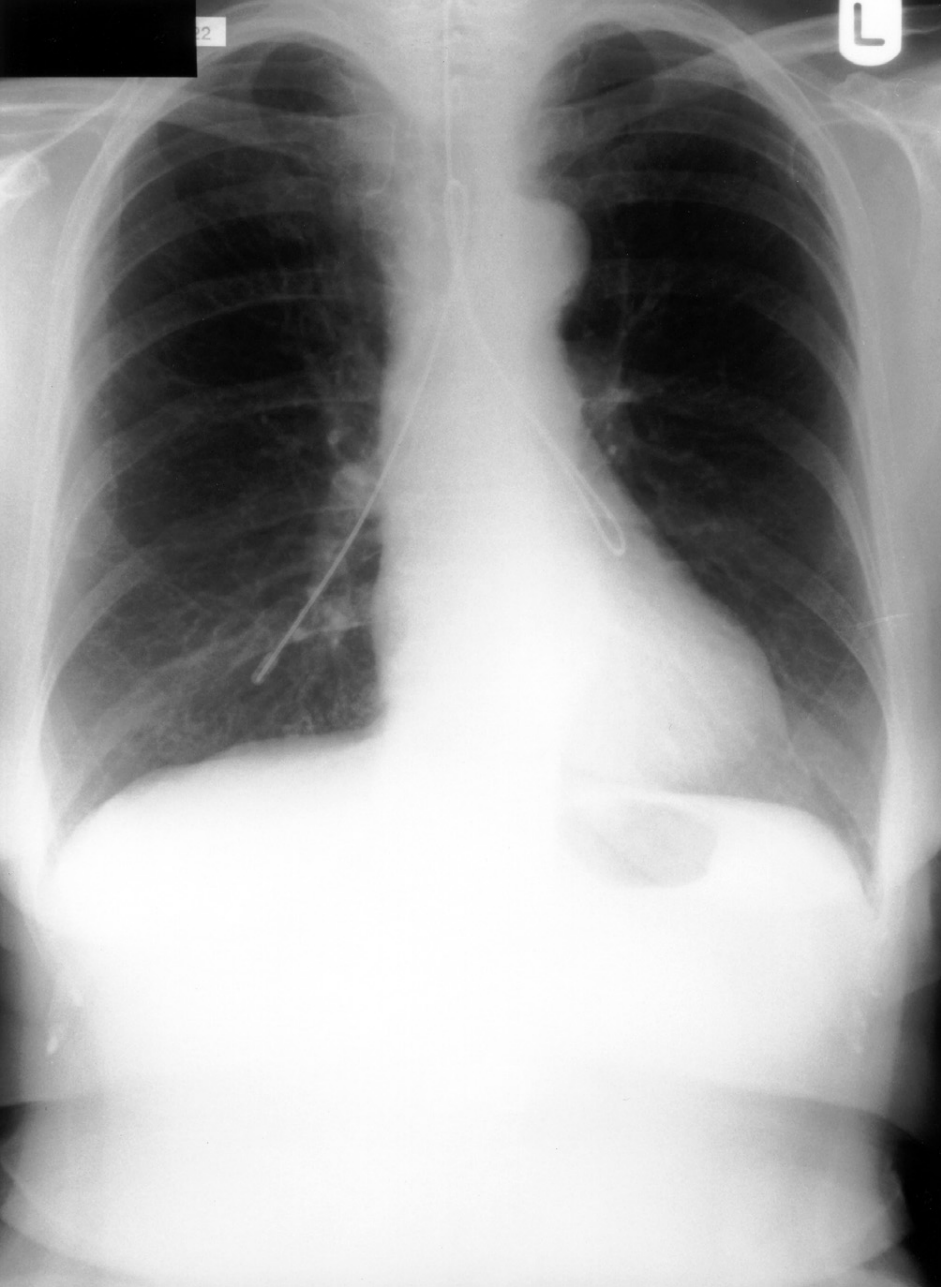
Chest x-ray showing a nasogastric tube folded into both main bronchi of a patient.

A 71-year old Caucasian lady presented to an Ear Nose and Throat (ENT) department with history of sore throat and odynophagia of six weeks duration. Endoscopy revealed an advanced hypopharyngeal carcinoma involving the posterior pharyngeal wall, both pyriform fossae and the postcricoid region. It was clinically staged as T3N2b postcricoid carcinoma. The histology showed it to be a poorly differentiated squamous cell carcinoma. The patient was treated by radiotherapy and neo-adjuvant chemotherapy over a six-week period. Following this treatment she was fed through a fine bore nasogastric tube and sent back to the ENT unit for recuperation. It was noticed that the tube was blocked. This was removed and attempts were made to insert a new fine bore tube with a stylet, and it was introduced up to the 50 cm mark without undue resistance. The patient, who was awake, coughed a few times during insertion but the coughing subsided spontaneously and rapidly. This therefore did not arouse any suspicion, and the procedure was deemed successful as confirmed by the auscultation of the epigastrium during insufflation of air. A check chest x-ray carried out immediately after tube replacement however showed the tube coiled in both main bronchi (Figure 1). Oesophagoscopy following the failed tube insertion showed a marked stenosis of the upper oesophagus, which was dilated. With the diagnosis of oesophageal stricture requiring repeated dilatations, the patient was discharged home on percutaneus endoscopic gastrostomy (PEG) tube feeding and was later weaned off after a few dilatations.

## Discussion

Nasogastric tube feeding is common practice in many clinical conditions. Many patients that receive these tubes are seriously ill, and have impaired cough and gag reflexes [3]. These patients are at increased risk of misplacement of nasogastric tubes. Patients with head and neck cancer pose a special problem as this may lead to upper aero-digestive tract obstruction or pain as a result of the disease and/or treatment. In a few cases, in order to bypass the structural obstruction, a general anaesthetic is required to pass the nasogastric tube. The tumour may also make it impossible to carry out a PEG and therefore an alternative route such as an open gastrostomy or gastrostomy under ultra sound guidance may be resorted to.

Nasogastric tube placement is usually uneventful. Pleural effusion, lung abscess and retropharyngeal abscess as a complication of malpositioned nasogastric tube have all been reported in the literature [4-7]. These patients may not present with the symptoms indicative of the malpositioning. The malposition was identified only after chest x-ray was taken or after tube feeding had begun, with resulting respiratory complications. Occasionally the malposition was missed or misinterpreted on chest x-ray [3,4]. In this patient who was awake and alert the nasogastric tube had travelled from one main bronchus to the other. It had bent over itself to finally lie in both main bronchi and this has not been previously reported in the literature. Auscultating the abdomen while insufflating air was false positive in this patient and therefore not a reliable method in confirming the correct position of a nasogastric tube. Insertion of a tube up to the usual recommended length of between 40 cm & 50 cm may also give a false sense of success, as was the case in this patient. However in selected cases, asking a patient (without definite obstruction of pharynx/oesophagus) to vocalize by saying “eeeee” in a high pitched note having inserted the tube to a reasonable level should close the laryngeal inlet allowing safe insertion of tube into the oesophagus [8].

## Conclusion

There was an apparent successful insertion of the tube in this patient until the chest x-ray showed bilateral bronchial intubation. Although there was no complication in this patient the potential for this is obvious. The case once again highlights the importance of taking a chest x-ray following nasogastric tube placement before feeding is commenced rather than relying on other confirmatory manoeuvres.

## List of abbreviations

ENT: Ear Nose and Throat
PEG: Percutaneus endoscopic gastrostomy
